# Boosting with an aerosolized Ad5-nCoV elicited robust immune responses in inactivated COVID-19 vaccines recipients

**DOI:** 10.3389/fimmu.2023.1239179

**Published:** 2023-10-04

**Authors:** Zhe Zhang, Shipo Wu, Yawei Liu, Kailiang Li, Pengfei Fan, Xiaohong Song, Yudong Wang, Zhenghao Zhao, Xianwei Zhang, Jin Shang, Jinlong Zhang, Jinghan Xu, Yao Li, Yaohui Li, Jipeng Zhang, Kefan Fu, Busen Wang, Meng Hao, Guanying Zhang, Pengwei Long, Ziyu Qiu, Tao Zhu, Shuling Liu, Yue Zhang, Fangze Shao, Peng Lv, Yilong Yang, Xiaofan Zhao, Yufa Sun, Lihua Hou, Wei Chen

**Affiliations:** ^1^ Beijing Institute of Biotechnology, Beijing, China; ^2^ Health Service Department of the Guard Bureau of the General Office of the Central Committee of the Communist Party of China, Beijing, China; ^3^ CanSino Biologics Inc., Tianjin, China

**Keywords:** Ad5-nCoV vaccine, aerosolized, SARS-CoV-2, BA.5 Omicron Variant, neutralizing Abs, saliva IgA

## Abstract

**Introduction:**

The SARS-CoV-2 Omicron variant has become the dominant SARS-CoV-2 variant and exhibits immune escape to current COVID-19 vaccines, the further boosting strategies are required.

**Methods:**

We have conducted a non-randomized, open-label and parallel-controlled phase 4 trial to evaluate the magnitude and longevity of immune responses to booster vaccination with intramuscular adenovirus vectored vaccine (Ad5-nCoV), aerosolized Ad5-nCoV, a recombinant protein subunit vaccine (ZF2001) or homologous inactivated vaccine (CoronaVac) in those who received two doses of inactivated COVID-19 vaccines.

**Results:**

The aerosolized Ad5-nCoV induced the most robust and long-lasting neutralizing activity against Omicron variant and IFNg T-cell response among all the boosters, with a distinct mucosal immune response. SARS-CoV-2-specific mucosal IgA response was substantially generated in subjects boosted with the aerosolized Ad5-nCoV at day 14 post-vaccination. At month 6, participants boosted with the aerosolized Ad5-nCoV had remarkably higher median titer and seroconversion of the Omicron BA.4/5-specific neutralizing antibody than those who received other boosters.

**Discussion:**

Our findings suggest that aerosolized Ad5-nCoV may provide an efficient alternative in response to the spread of the Omicron BA.4/5 variant.

**Clinical trial registration:**

https://www.chictr.org.cn/showproj.html?proj=152729, identifier ChiCTR2200057278.

## 1 Introduction

More than 6.8 million people have died from COVID-19 worldwide since the start of the pandemic (1). The COVID-19 vaccines studied to date are highly effective against severe disease and death. However, immunity from the COVID-19 vaccines is waning, and variants capable of different degrees of immune evasion are continuously emerging (2–4); thus, there is a clear and urgent need for booster vaccination to increase vaccine effectiveness against severe disease and death.

More than 100 countries worldwide have already issued recommendations on booster or additional vaccination (5). Both homologous and heterologous booster regimens including mRNA vaccines, adenovirus-vectored vaccines, inactivated vaccines and recombinant protein vaccines are immunologically effective, and no safety issues have been observed. In Israel, a booster dose of the BNT162b2 vaccine induced a more than 10-fold decrease in the relative risk of confirmed infection and severe illness compared with that of the nonbooster group (6). The effectiveness of a booster dose of the BNT162b2 vaccine reached 93% for admission to the hospital compared with that upon receipt of only two doses at least 5 months prior (7).

In China, seven COVID-19 vaccines have been authorized for use, including five inactivated vaccines, an adenovirus-vectored vaccine (Ad5-nCoV, Convidecia) and a recombinant protein subunit vaccine (ZF2001, Zifivax). These vaccines have been shown to be efficacious in preventing mild to severe COVID-19 (8–10). Additionally, Ad5-nCoV has recently been proven to induce a robust humoral and cellular immune response through inhaling immunization pathway both in the prime and boost vaccination (11, 12). To date, more than 3.4 billion doses of COVID-19 vaccines have been administered in China, and over 95% of these doses were of the inactivated vaccines. As of May, 2022, more than 770 million individuals have received booster vaccination in China, 95% of whom received homologous booster vaccination (13).

Booster vaccination strategies based on inactivated vaccine priming have been well studied, and heterologous vaccination regimens induce immune responses that are superior to those induced by homologous regimens (14–17). Clemens et al. reported that the increases in specific IgG titers from baseline to 28 days were 12-fold for CoronaVac (the inactivated vaccine), 152-fold for BNT162b2, 90-fold for ChAdOx1 nCoV-19, and 77-fold for Ad26.COV2.S after booster vaccination in CoronaVac-primed recipients (16). Li et al. reported that neutralizing antibody titers were increased by 79-fold for Ad5-nCoV booster vaccination and by 15-fold for CoronaVac booster vaccination from before booster vaccination to day 14 after booster vaccination in subjects who received two doses of CoronaVac (17).

To optimize the booster vaccination regimen in persons who have received two doses of inactivated vaccines in China, we performed a head-to-head immunological comparison of the COVID-19 vaccines available in China, including intramuscular Ad5-nCoV, aerosolized Ad5-nCoV, a recombinant protein subunit vaccine (ZF2001) and homologous CoronaVac booster administration in inactivated vaccine-primed recipients who were vaccinated 6 months prior.

## 2 Materials and methods

### 2.1 Study design

This study was a non-randomized, open-label and parallel-controlled phase 4 trial (ChiCTR2200057278). 904 eligible participants were randomly assigned to four groups using computer-generated random numbers in SPSS, to receive one dose of Ad5-nCoV via intramuscular injection (Ad5-nCoV-IM, 5×10^10^ viral particles), aerosolized Ad5-nCoV (Ad5-nCoV-IH, 1×10^10^ viral particles), a recombinant protein subunit vaccine (ZF2001, 25 μg) or an inactivated vaccine (CoronaVac, 3 μg) in December 2021. Only laboratory staff were masked to group assignments. All participants who received two doses of inactivated vaccine (65.4% receiving CoronaVac, 23.3% receiving BBIBP-CorV (Sinopharm) and 11.3% receiving the mixed CoronaVac and BBIBP-CorV) 6 months prior were enrolled in this study. Participants were excluded if they were previously infected with COVID-19 or had an immunosuppressive condition by self-reporting. Participants were followed longitudinally to evaluate the immune response to different boosters at days 0, 7, 14, 28 and months 3 and 6 after booster vaccination. During the recruiting, 904 eligible participants were assigned a number from 0 to 904. In each group, participants were sorted from smallest to largest by their number. The first 50 participants in each group were selected for more in-depth immunological analysis of mucosal IgA, neutralizing antibody, and cellular response. All the participants provided written informed consent to take part in the study. The protocol was approved by the Ethics Committee of 305 Hospital of PLA.

### 2.2 Vaccines

All COVID-19 vaccines in this study were developed based on the SARS-CoV-2 wild-type strain (Wuhan-Hu-1 strain) isolated in December 2019. Ad5-nCoV (Convidecia) is a replication-defective human type 5 adenovirus-vectored vaccine, encoding the full-length Spike gene of the Wuhan-Hu-1 strain (YP_009724390) (10). Participants in Ad5-nCoV-IM group received the intramuscular Ad5-nCoV at 0.5 mL per dose. The aerosolized Ad5-nCoV is the same vaccine as Ad5-nCoV that is administered via oral inhalation (11). Participants orally inhaled the aerosolized droplets of Ad5-nCoV at 0.1 mL per dose in a disposable suction cup, which is aerosolized by a continuous vapouring system (which contained a vaporing unit [Aerogen, Galway, Ireland] integrated by Suzhou Weiqi Biological Technology [Suzhou City, China]). ZF2001 is a recombinant tandem-repeat dimeric RBD-based protein subunit vaccine, which is expressed in a CHO cell system (18). Participants received intramuscular ZF2001 at 0.5 mL (25 μg) per dose. CoronaVac is an inactivated vaccine from the SARS-CoV-2 strains isolated during the initial outbreak in China (19). Participants were administered intramuscularly at 0.5 mL (3 μg) per dose.

### 2.3 RBD-binding IgG assay

RBD-binding IgG antibodies in the heat-inactivated human serum samples and the culture supernatant of PBMCs stimulated for 4 days with R848 + IL-2 were detected with an RBD-binding IgG ELISA kit (Beijing, Kewei). Briefly, diluted samples and a reference standard were added in duplicate to rSARS-CoV-2 RBD-precoated wells and incubated for 30 min at 37°C. The microplates were washed, and a horseradish peroxidase (HRP)-conjugated goat anti-human IgG secondary antibody was added to bind the RBD-bound human antibodies. After 30 min of incubation, the microplates were washed, and TMB chromogenic substrate was added to generate a colorimetric signal for 10 min. A stop solution was added to stop color development, and the signal was read on a microplate reader. The total anti-RBD IgG antibody levels were quantitated in ELISA units (EU) ml^-1^ by comparison to a reference standard curve created from monoclonal antibodies against SARS-CoV-2 RBD. The results were analyzed by GraphPad Prism 8.0.2 using 4-PL curve fitting. Seroconversion was defined as at least a 4-fold increase in concentration of IgG from baseline to post-vaccination as described previously (20). The WHO international standard for anti-SARS-CoV-2 IgG (NIBSC code 20/136) was used as a reference.

### 2.4 Coronavirus-specific saliva IgA assay

Saliva samples were collected by centrifugation after having each subject spit about 2 mL of saliva into a disposable saliva sample collector (HUAXIA Medical Equipment). Coronavirus-specific IgA was tested with a SARS-CoV-2 specific (COVID-19 Coronavirus Panel 2) Mesoscale Discovery (MSD) immunoassay. According to the manufacturer’s instructions, plates were blocked, washed, and incubated with diluted saliva samples. After incubation with the detection antibody, plates were read on a Meso QuickPlex instrument. IgA concentrations were showed in arbitrary units (AU) per ml as calculated from a standard curve supplied with the kit. To minimize the variability of mucosal sampling, saliva samples were normalized via an enzyme-linked immunosorbent assay for quantitative detection of human IgA using a commercial kit (Invitrogen) as described previously (21). The levels of coronavirus-specific saliva IgA in participants were normalized per μg of their total saliva IgA.

### 2.5 Pseudotype-based neutralization assays

The generation of SARS-CoV-2 Pseudoviruses was performed using the human immunodeficiency virus (HIV) pseudotyped virus production system (22). HEK293T cells were cotransfected with the pNL4.3-Luc-R^-^E^-^ and the indicated pCAGGS-based plasmids with TurboFect transfection reagent (Thermo Scientific). The pCAGGS-S^WT^, pCAGGS-S^BA.1^, pCAGGS-S^BA.4/5^, pCAGGS-S^BQ.1.1^, pCAGGS-S^XBB^, and pCAGGS-S^CH.1.1^ plasmids were constructed and encoded the wild-type (hCoV-19/Wuhan/Hu-1/2019, GISAID EPI_ISL_402125), the Omicron BA.1 variant (hCoV-19/Botswana/R40B60_BHP_3321001247/2021, GISAID EPI_ISL_6640917), the Omicron BA.4 variant (hCoV-19/South Africa/NICD-N35214/2022, GISAID EPI_ISL_11542270), the Omicron BQ.1.1 variant (hCoV-19/England/PHEC-YYGA6S6/2022, GISAID EPI_ISL_14919574), the Omicron XBB variant (hCoV-19/USA/NY-PRL-220907_01F03/2022, GISAID EPI_ISL_14914850), and the Omicron CH.1.1 variant (hCoV-19/USA/GA-CDC-STM-DR6ZRD6VB/2022, GISAID EPI_ISL_15529969) of the SARS-CoV-2 virus spike glycoprotein, respectively. Supernatants were collected 48 hours posttransfection, filtered, aliquoted and frozen at −80°C before use.

Neutralizing activity in each sample was measured with a serial dilution approach as Nie et al. reported (23). Each sample was serially diluted 3-fold in duplicate from 1:30 to 1:7290 or 1:21870 in complete DMEM before incubation with the titrated pseudovirus SARS-CoV-2 for 1 hour prior to the addition of 2×10^4^ 293T-ACE2 cells. Following a 48-h incubation period at 37°C and 5% CO_2_, luciferase activity was determined with the BriteLite Plus Reporter Gene Assay System (PerkinElmer) using a microplate reader (Tecan). EC_50_ neutralization titers were calculated using the Reed-Muench method. The lower limit of detection (LLOD) was 30, and titers below the LLOD were set to 15. The WHO international standard for anti-SARS-CoV-2 IgG (NIBSC code 20/136) was used as a reference.

### 2.6 RBD-ACE2 competitive binding assay

The RBD neutralizing antibody against the different SARS-CoV-2 variants was evaluated using a commercial ELISA kit (Vazyme) by a surrogate virus neutralization test (sVNT). Sera are serially diluted by 3-fold from 1:5 to 1:1215 or 1:10935 with horseradish peroxidase-labeled recombinant RBD (HRP-RBD) protein and incubated at 37 °C for 30 minutes. HRP-RBD without the serum were added in duplicated in each plate as the negative control. Then 100 μl of dilution mixture was transferred to the corresponding wells of microplate that precoated hACE2 protein. After the incubation at 37 °C for 20 minutes, each well was washed with 350μl of wash buffer for 4 times. 100 μl of TMB substrate was added and incubated for 15 minutes at 37 °C and 100 μl of stop solution was used to stop the reaction, immediately followed by the plate reading at 450nm. The antibody titer was calculated as the reciprocal of the dilution for which the OD value was reduced by 50% of that of the negative control (IC_50_) using nonlinear regression with four parameters in GraphPad Prism 8.0.

### 2.7 Spike-specific IgG ELISpot assays

Spike-specific IgG ELISpot assays were performed on R848- and IL-2-activated PBMCs with a human IgG ELISpot Kit (Mabtech). Briefly, fresh PBMCs were activated with a mixture of R848 at 1 μg ml^-1^ and rhIL-2 at 10 ng ml^-1^ for 4 days. PVDF ELISpot plates were coated with a purified anti-human IgG monoclonal antibody (MT91/145), incubated at 4-8°C overnight, and blocked with RPMI 1640 medium containing 10% fetal bovine serum and 1× penicillin–streptomycin solution (Gibco) for at least 30 min at room temperature. Activated PBMCs were washed to remove any secreted antibodies, counted, diluted to the indicated concentration, and added to the ELISpot plates. Tests were performed in duplicate, with 20,000-200,000 cells per well in 100 μl of medium. The plates were incubated in a 37°C humidified incubator with 5% CO_2_ for 16-24 hours. The secretion of spike-specific IgG was visualized by the addition of a biotinylated ancestral SARS-CoV-2 spike antigen (1 μg ml^-1^) followed by streptavidin-HRP and TMB substrate. The SARS-CoV-2 spike antigen (Vazyme) was labeled using an EZ-link NHS-PEG4-Biotin (Thermo Fisher), then purified through Zeba Spin Desalting Columns (Thermo Fisher) and quantified by a Pierce BCA Protein Assay Kit (Thermo Fisher). The spots were counted using an ELISpot counter (SinSage Technology), and the results are expressed as SARS-CoV-2 spike-specific IgG spot-forming cells (SFCs) per 10^6^ PBMCs.

### 2.8 IFNγ ELISpot assays

IFNγ ELISpot assays were performed with fresh PBMCs and a human IFNγ ELISpot Kit (Mabtech) following the manufacturer’s instructions. Tests were performed in duplicate. The precoated ELISpot plates were washed with sterile PBS and blocked with RPMI 1640 medium containing 10% fetal bovine serum and 1× penicillin–streptomycin solution (Gibco) for at least 30 min at room temperature. Fresh PBMCs were added at 2 × 10^5^ cells per well along with an overlapping 15-amino-acid peptides pool covering the ancestral SARS-CoV-2 spike protein (GL Biochem (Shanghai) Ltd.) (1 μg ml^-1^/peptide) or the same volume of DMSO for unstimulated controls. The cells were incubated in a 37°C humidified incubator with 5% CO_2_ for 16-24 hours. IFNγ spots were detected after the addition of a biotinylated detection antibody (7-B6-1-biotin, 1 μg ml^−1^) followed by streptavidin-HRP and TMB substrate. The spots were counted using an ELISpot counter (SinSage Technology). The counts were summarized as the mean values of duplicate wells with the values of the unstimulated wells subtracted, and negative values were set to zero. The results are expressed as SARS-CoV-2 spike-specific IFNγ SFCs per 10^6^ PBMCs. Responses were considered positive if there were ≥50 spike-specific SFCs per 10^6^ PBMCs as reported before (20), and the ratio of spots in the stimulated wells to spots in the background wells was no less than 2.1.

### 2.9 Statistical analysis

All analyses of participant samples were conducted using GraphPad Prism 8.0.2 or SAS 9.4. Levels of antibodies against SARS-CoV-2 are presented as the median concentration or median titer with IQR. Spike-specific IgG spots and IFNγ responses are depicted as the median with IQR. Categorical data were analyzed by the χ^2^ test or Fisher’s exact test. Multiple group comparisons were analyzed by running a nonparametric (Kruskal-Wallis test) statistical test and corrected using Dunn test as indicated in the figure legends. The correlation between concentrations of log-transformed neutralizing antibody and binding antibody levels was analyzed using Spearman’s correlation. P values less than 0.05 were considered to indicate statistical significance.

## 3 Results

### 3.1 Baseline characteristics of the participants

**Figure 1:**
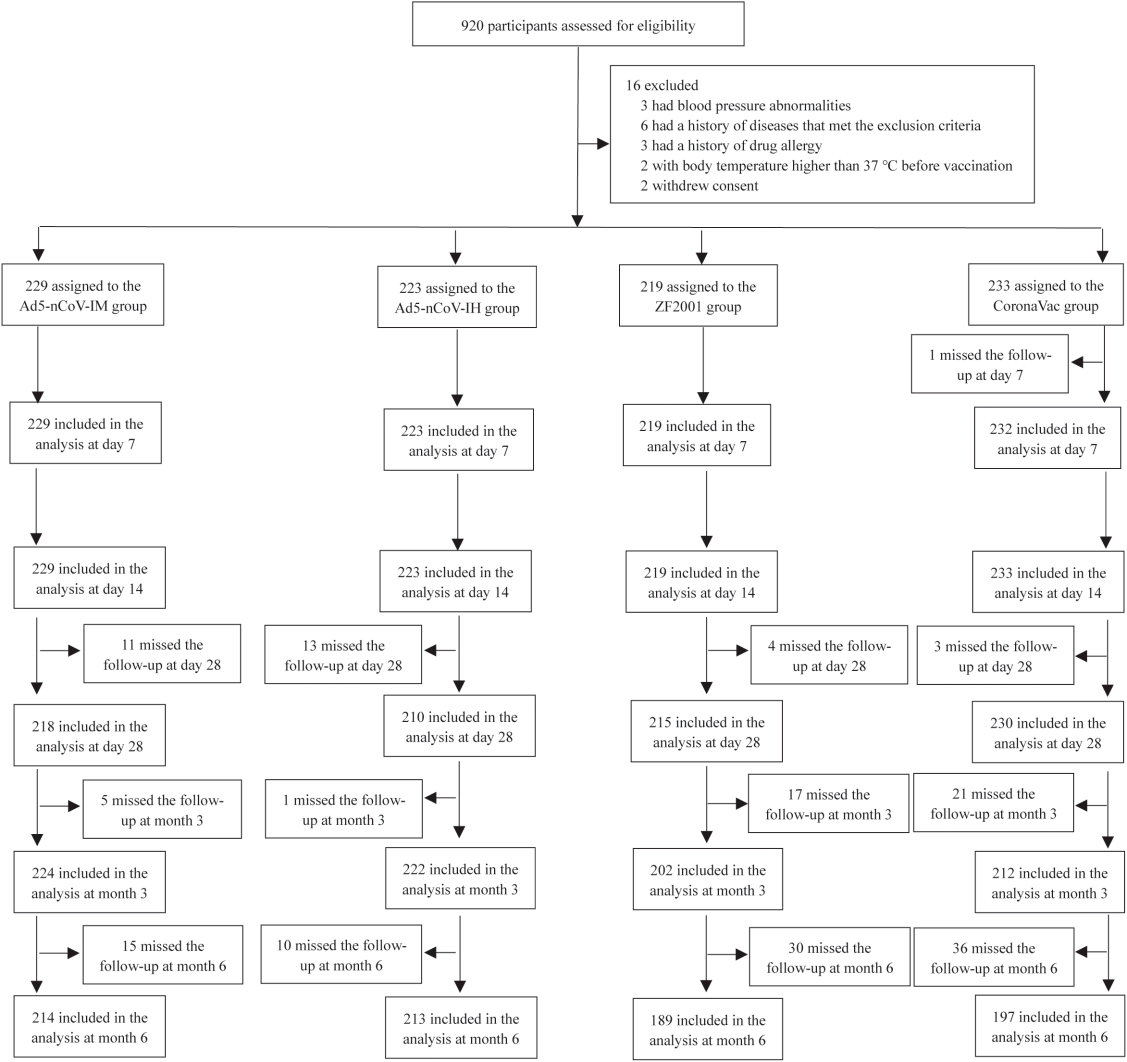
Trial profile.

In this study, 904 subjects who received two doses of inactivated vaccine 6 months prior were assigned to 4 groups for booster vaccination (**Figure 1**). The participants in two of the groups received Ad5-nCoV booster vaccination by different delivery routes. A total of 229 participants in the Ad5-nCoV-IM group were intramuscularly vaccinated with 5×10^10^ viral particles of Ad5-nCoV per dose, and 223 participants in the Ad5-nCoV-IH group were vaccinated with 1×10^10^ viral particles of aerosolized Ad5-nCoV per dose. A total of 219 participants in the ZF2001 group received the recombinant protein subunit vaccine (ZF2001), and 233 participants in the CoronaVac group received an inactivated vaccine (CoronaVac). The baselines of the participants, including age, sex, interval to prime vaccination, and anti-SARS-CoV-2 neutralizing antibodies, were comparable among the four groups (**Table 1**).

**Table 1:** Baseline characteristics of the participants.

	Ad5-nCoV-IM Group	Ad5-nCoV-IH Group	ZF2001 Group	CoronaVac Group
**Participants number**	229	223	219	233
Age in years				
Median (Min, Max)	21.0 (19.0, 25.0)	21.0 (19.0, 25.0)	21.0 (19.0, 25.0)	21.0 (19.0, 24.0)
Sex (%)				
Male	229 (100.0)	223 (100.0)	219 (100.0)	233 (100.0)
Female	0 (0.0)	0 (0.0)	0 (0.0)	0 (0.0)
Baseline neutralizing antibody against SARS-CoV-2				
Sample number	48	48	49	51
Median titer (IQR)	15 (15-15)	15 (15-15)	15 (15-15)	15 (15-15)
Seropositivity (%)	8.3	6.3	8.2	11.8
The interval between the last priming dose of inactivated vaccine and the booster (months)				
Median (IQR)	6.3 (6.0, 6.8)	6.2 (6.0, 6.5)	6.2 (6.0, 6.7)	6.2 (6.0, 6.7)
The distribution ratios of priming regimen (BBIBP-CorV; CoronaVac; a combination of both)				
	23.6%; 65.9%; 10.5%	23.3%; 66.4%; 10.3%	25.6%; 59.8%; 14.6%	21.0%; 69.1%; 9.9%

Data are n (%) or median. Seropositivity for neutralizing antibody against SARS-CoV-2 from a subset of participants in each group before receiving a booster vaccination at day 0 is defined as a detectable neutralizing antibody titer ≥ 1:30.

### 3.2 SARS-CoV-2-specific serum IgG and mucosal IgA responses

**Figure 2:**
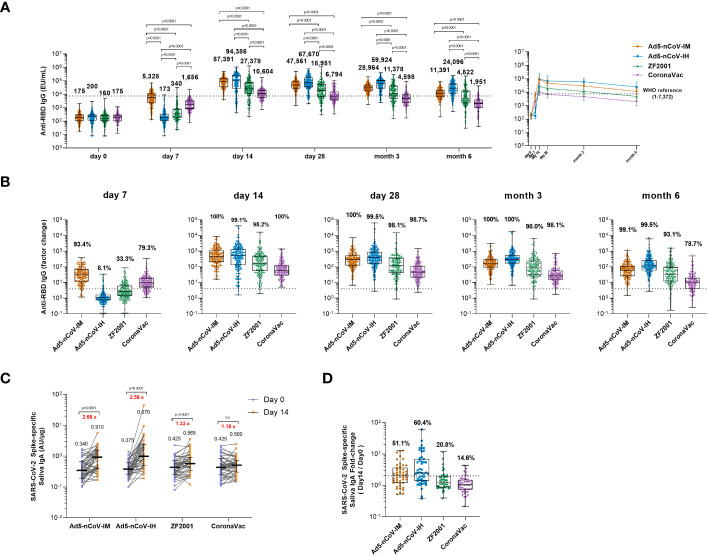
Wild-type SARS-CoV-2 RBD-specific binding antibody responses. **(A)** The concentrations of SARS-CoV-2 RBD-specific IgG antibodies at day 0 (before booster vaccination) and days 7, 14, 28 and months 3 and 6 after booster vaccination in the four groups. The numbers above the bars are median concentrations for each group, and connecting lines reflect median concentration. The dashed line indicates the WHO reference (1,000 binding antibody units (BAU) ml^-1^ in serum) which is equivalent to an RBD-specific IgG antibody titer of 1:7,372. Statistical significance was determined by Kruskal–Wallis test with Dunn’s multiple comparisons tests. **(B)** Per-participant factor changes that were calculated by dividing the after-booster response by the before-booster response for RBD-specific binding antibodies. The dashed line indicates a factor change of 4 (the lower limit of seroconversion), and the number above the dashed line indicates the seroconversion of RBD-specific IgG responses. **(C)** The SARS-CoV-2 Spike-specific saliva IgA concentrations on day 0 and day 14 after the booster. Error bars indicate IQR, and the black numbers above the points are normalized saliva IgA median concentrations. Red numbers on the top are the fold-changes of IgA median concentrations from day 0 to day 14. Statistical significance was determined by Wilcoxon’s signed rank test. **(D)** Per-participant factor changes that were calculated by dividing the after-booster response by the before-booster response for saliva IgA. The dashed line indicates a factor change of 2, and the number above the dashed line indicates the proportion of the participants whose IgA level has increased at least twice. For **(A, B, D)** the whiskers indicate the range, the top and bottom of the boxes indicate the interquartile range, and the horizontal line within each box indicates the median.

Concentrations of anti-SARS-CoV-2 IgG and IgA antibodies were assessed before and after the boost. (**Figures 2A**, **S1**). In all groups, IgG antibody concentrations peaked on 14 days and dropped slightly after 28 days post-vaccination. Intramuscular injection of Ad5-nCoV elicited the most significant and rapid increase in anti-RBD IgG antibodies by 30-fold compared to baseline, followed by CoronaVac, with a 9-fold increase compared to baseline at 7 days (**Figure 2A**). ZF2001 booster vaccination slightly enhanced anti-RBD IgG antibodies, with a 2-fold increase compared to baseline, whereas the administration of aerosolized Ad5-nCoV did not alter the anti-RBD IgG antibody levels. The seroconversion rate reached 93.4% for Ad5-nCoV-IM and 79.3% for CoronaVac (**Figure 2B**). After 14 days, the magnitude of the IgG response reached to peak in all the groups. All heterologous regimens were superior to homologous CoronaVac booster vaccination (P<0.0001), and both intramuscular and aerosolized Ad5-nCoV vaccination induced similarly increases in the IgG response compared to that of ZF2001 (P<0.0001). At day 28, the anti-RBD IgG antibody response decreased slightly in all the groups compared to day 14, except for the Ad5-nCoV-IH. The median concentration of IgG in the Ad5-nCoV-IH group was higher than those in the ZF2001 and CoronaVac groups (P<0.0001), and similar to that in the Ad5-nCoV-IM group. At month 6, the anti-RBD IgG antibody median concentration decreased by 4.2, 2.8, 3.9 and 3.5 times in Ad5-nCoV-IM, Ad5-nCoV-IH, ZF2001 and CoronaVac groups respectively compared to day 28 and the median concentration of Ad5-nCoV-IH group was significantly higher than those of Ad5-nCoV-IM (P<0.0001), ZF2001 (P<0.0001) and CoronaVac group (P<0.0001). The seroconversion rate in CoronaVac group decreased to 78.7% while those in other three groups were still higher than 93%.

The level of saliva IgA at day 0 and 14 after boost was also measured to evaluate the mucosal immune response induced by different vaccines. An obvious increase in IgA response to spike at day 14 was observed in intramuscular Ad5-nCoV (2.68-fold, p<0.001), aerosolized Ad5-nCoV (2.59-fold, p<0.001) and ZF2001 (1.33-fold, p=0.0027) recipients compared with that in CoronaVac recipients (**Figure 2C**). 60.4% of the participants in Ad5-nCoV-IH group showed an improvement of IgA level at least twice over the baseline, followed by 51.1% in Ad5-nCoV-IM, 20.8% in ZF2001 and 14.6% in CoronaVac group (**Figure 2D**).

These data suggest that heterologous boosting with Ad5-nCoV via different routes can elicit significantly higher RBD-specific IgG levels than ZF2001 or CoronaVac, and the aerosolized Ad5-nCoV induced a distinct mucosal immune response.

### 3.3 Pseudotyped SARS-CoV-2 neutralizing antibody responses

**Figure 3:**
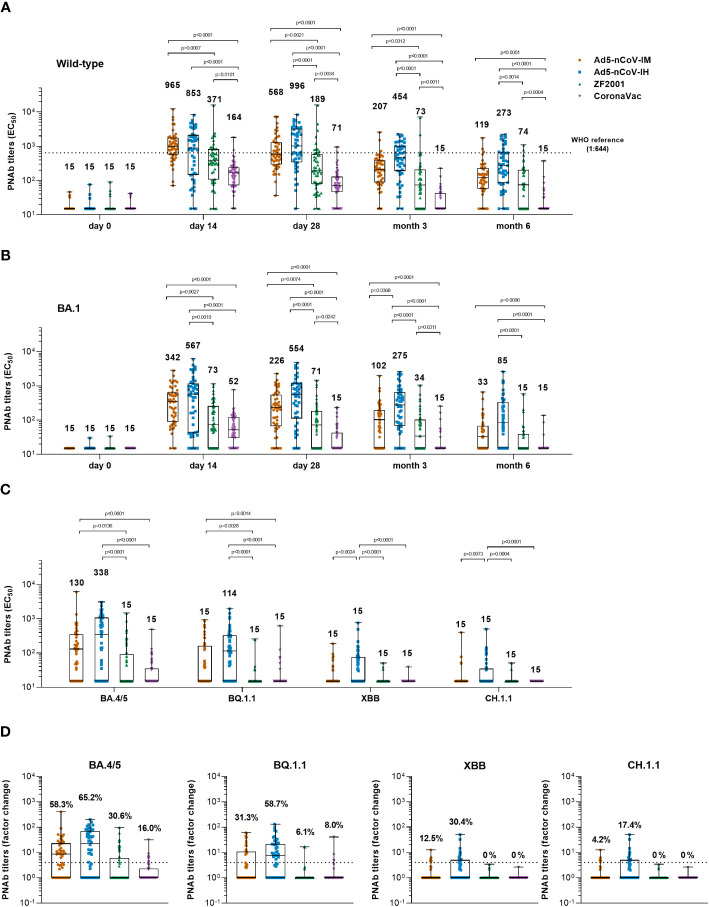
Pseudotyped SARS-CoV-2 neutralizing antibody responses. **(A, B)** Median titers of PNAb to wild-type SARS-CoV-2 **(A)** or the Omicron variant **(B)** at days 0 (before booster vaccination), 14, 28 and months 3 and 6 after booster vaccination in the four groups. The numbers on the top of the bars are median titers for the group. The dashed line indicates the WHO reference (1,000 binding antibody units (BAU) ml^-1^ in serum) which is equivalent to a PNAb titer of 1:644 to the wild-type and is lower than the limit of detection to the Omicron BA.1 variant of SARS-CoV-2. **(C)** Median titers of SARS-CoV-2 Omicron variants including BA.4/5, BQ.1.1, XBB and CH.1.1 at day 28 after booster vaccination in the four groups. The numbers on the top of the bars are median titers for the group. **(D)** Per-participant factor changes that were calculated by dividing the after-booster response by the before-booster titer for PNAb. The number above the dashed line indicates the seroconversion of PNAb responses. The whiskers indicate the range, the top and bottom of the boxes indicate the interquartile range, and the horizontal line within each box indicates the median. Statistical significance was determined by Kruskal–Wallis test with Dunn’s multiple comparisons tests.

Before booster vaccination, only 6.3%~11.8% of participants had a weak wild-type pseudovirus neutralizing antibody (PNAb) titer. Generation of PNAb against wild-type SARS-CoV-2 was significantly increased after booster vaccination in all groups (**Figure 3A**). At day 14, participants who received intramuscular Ad5-nCoV had the highest median titer of PNAb at 965 (Interquartile Range, IQR = 565-1706), compared with a median titer of 853 (IQR = 144-2070) in the Ad5-nCoV-IH group, a median titer of 371 (IQR = 105-802) in the ZF2001 group (P=0.0007) and a median titer of 164 (IQR = 71-235) in the CoronaVac group (P<0.0001). An increased PNAb response was also observed when heterologous aerosolized Ad5-nCoV (P<0.0001) or ZF2001 (P=0.0101) was compared with the homologous CoronaVac. At day 28, the PNAb level in the Ad5-nCoV-IH group slightly differed from its IgG response, as an increased median titer of 996 (IQR = 330-3116) was observed, while those in the Ad5-nCoV-IM, ZF2001 and CoronaVac groups decreased to 568 (IQR = 287-1256), 189 (IQR = 77-574) and 71 (IQR = 46-128), respectively. At month 6, PNAb level decreased by 2.6-4.8 times in all the groups compared to day 28, with the highest median titer of 273 (IQR = 84-637) in the Ad5-nCoV-IH group, and the lowest median titer of 15 (IQR = 15-15) in the CoronaVac group.

Similar kinetics were observed for the PNAb response against the Omicron BA.1 variant (**Figure 3B**). Both the Ad5-nCoV-IM and Ad5-nCoV-IH groups exhibited a remarkably higher PNAb level than the other two groups from day 14 to month 6. Specifically, participants in the Ad5-nCoV-IH group generated the most robust response at day 28, with a median titer of 554 (IQR = 111-1178), followed by the Ad5-nCoV-IM group with a median titer of 226 (IQR = 66-545), the ZF2001 group with a median titer of 71 (IQR = 15-181, P<0.0001) and the CoronaVac group with a median titer of 15 (IQR = 15-43, P<0.0001). The lowest seroconversion rate of 18.0% was observed in CoronaVac group (**Figure S2**). After 6 months, the highest PNAb response was generated by aerosolized Ad5-nCoV (median titer = 85, IQR = 15-332), which was at 5.7-fold that of CoronaVac (median titer = 15, IQR = 15-15, P<0.0001), 5.7-fold that of ZF2001 (median titer = 15, IQR = 15-40, P<0.0001) and 2.6-fold that of intramuscular Ad5-nCoV (median titer = 33, IQR = 15-67). The best correlation between RBD-IgG and PNAb against Omicron BA.1 was in Ad5-nCoV-IH group while the worst was in CoronaVac group (**Figure S3**).

The neutralization activity against new-emerging Omicron subvariants including BA.4/5, BQ.1.1, XBB and CH.1.1 was further evaluated (**Figures 3C, D**). At day 28, participants in Ad5-nCoV-IH group had the highest median titer and seroconversion rate of neutralizing antibodies. The median titer of BA.4/5 and BQ.1.1-specific PNAb was 338 (IQR = 15-1055) and 114 (IQR = 15-326) in Ad5-nCoV-IH group, followed by 130 (IQR = 15-329) and 15 (IQR = 15-114) in Ad5-nCoV-IM, 15 (IQR = 15-92, P<0.0001) and 15 (IQR = 15-15, P<0.0001) in ZF2001, 15 (IQR = 15-35, P<0.0001) and 15 (IQR = 15-15, P<0.0001) in CoronaVac group (**Figure 3C**). The neutralization of XBB and CH.1.1 by sera was markedly impaired. The PNAb response against XBB and CH.1.1 induced by aerosolized Ad5-nCoV sharply decreased to a level with the median titer of 15 (IQR = 15-76) and 15 (IQR = 15-35), respectively, although it was still higher than those induced by other boosters. Participants in Ad5-nCoV-IH group showed the seroconversion rates of PNAb against different Omicron subvariants ranging from 17.4% to 65.2% (**Figure 3D**). At month 6, the seroconversion rate of BA.4/5 specific PNAb reached 19.6%, 61.7%, 12.2% and 2.3% in the Ad5-nCoV-IM, Ad5-nCoV-IH, ZF2001 and CoronaVac group, respectively (**Figure S2C**). PNAb median titer against Omicron BA.4/5 variant decreased by 3.3-7.9 times in participants boosted with Ad5-nCoV or ZF2001 compared to those of wild-type strain (**Figure S4**). These results demonstrate that aerosolized Ad5-nCoV could provide a robust neutralizing antibody response against Omicron BA.1 and BA.4/5 variants, while it was highly resisted by the new subvariants XBB and CH.1.1.

### 3.4 SARS-COV-2 surrogate virus neutralization antibody response

**Figure 4:**
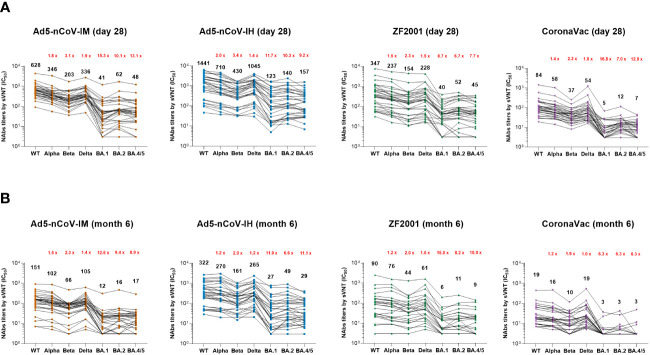
SARS-CoV-2 sVNT neutralizing antibody responses. **(A, B)** Median titers of NAbs against wild-type SARS-CoV-2 and the various variants at day 28 **(A)** and month 6 **(B)** after the booster by an RBD-ACE2 competitive binding assay. The connecting lines between the variants represent matched serum samples. Black numbers on the top are median titers for each variant. Red numbers on the top are the fold decline in median titers from the wild-type to the indicated variant of SARS-CoV-2. 30 participants in each group were tested.

The breadth and magnitude of neutralizing antibody responses to various SARS-CoV-2 variants were investigated via an SARS-CoV-2 Surrogate Virus Neutralization Test (sVNT) based on the RBD-ACE2 competitive binding assay. In all the groups, the better antibody responses were against wild-type strain, Alpha and Delta variants, and the worst were against Omicron variants. Ad5-nCoV-IH booster vaccination elicited most potent cross-reactivity neutralizing antibody responses in wild-type, Alpha, Beta, Delta, Omicron BA.1, BA.2 and BA.4/5 variants, followed with Ad5-nCoV-IM, ZF2001 and CoronaVac booster vaccination at day 28 and month 6 post-vaccination (**Figure 4**). The Nab median titer against each SARS-CoV-2 variant at day 28 after the booster decreased by 1.8-15.3, 1.4-11.7, 1.5-8.7 and 1.4-16.8 times in Ad5-nCoV-IM, Ad5-nCoV-IH, ZF2001 and CoronaVac group compared to those against the wild-type strain (**Figure 4A**). At month 6, participants boosted with the aerosolized Ad5-nCoV showed the highest neutralization antibody responses to SARS-CoV-2 wild-type and the variants among all the boosters, which were even similar to the responses induced by ZF2001 at day 28 after the booster (**Figure 4B**). sVNT neutralization antibody responses against wild-type and Omicron variant were substantially correlated with PNAb responses in all groups, especially in the Ad5-nCoV-IH group (**Figure S5**). Together with the PNAb responses, these results indicated that the aerosolized Ad5-nCoV can induce a robust and long-lasting neutralizing antibody response against SARS-CoV-2.

### 3.5 Spike-specific IgG B-cell responses

**Figure 5:**
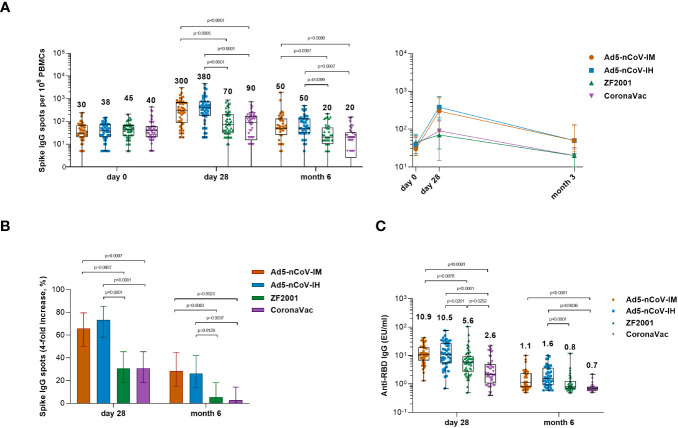
Wild-type SARS-CoV-2 spike-specific IgG B-cell responses. **(A)** Median numbers of SARS-CoV-2 spike-specific IgG spots at days 0 (before booster vaccination), 28 and month 6 after booster vaccination in the four groups. **(B)** Percentage of participants with a fourfold increase in spike-specific IgG spots. Error bars indicate 95% CIs. Statistical significance was determined by two-sided χ^2^ tests or Fisher’s exact test. **(C)** Median concentrations of SARS-CoV-2 RBD-specific IgG antibodies in the culture supernatant of R848- and IL-2-activated PBMCs at day 28 and month 6 after booster vaccination. For **(A, C)** the whiskers indicate the range, the top and bottom of the boxes indicate the interquartile range, and the horizontal line within each box indicates the median. The numbers above the bars are median concentrations for each group, and connecting lines reflect median concentration. Statistical significance was determined by Kruskal–Wallis test with Dunn’s multiple comparisons tests.

To further investigate the ability of the boosters to induce antigen-specific B-cell responses, spike-specific IgG spots were detected at baseline and at 28 days and 6 months after booster vaccination in R848-activated peripheral blood mononuclear cells (PBMCs) (**Figure 5**). Significantly more spike-specific IgG spots were detected in all groups at day 28 after booster vaccination (**Figure 5A**). The Ad5-nCoV vaccine induced a significant increase in the spike-specific IgG spot response in the Ad5-nCoV-IM group versus either the ZF2001 group (P=0.0005) or the CoronaVac group (P<0.0001) and the Ad5-nCoV-IH group versus either the ZF2001 group (P<0.0001) or the CoronaVac group (P<0.0001). The median number of IgG spots per 10^6^ PBMCs at day 28 was 300 (IQR, 85-670) for Ad5-nCoV-IM, 380 (IQR, 175-730) for Ad5-nCoV-IH, 70 (IQR, 30-200) for ZF2001 and 90 (IQR, 15-165) for CoronaVac; these values changed to 50 (IQR, 25-130), 50 (IQR, 30-128), 20 (IQR, 10-53) and 20 (IQR, 3-33) at month 6. A total of 65.9% (95% CI, 50.1%-79.5%) and 28.2% (95% CI, 15.0%-44.9%) of participants in the Ad5-nCoV-IM group and 73.3% (95% CI, 58.1%-85.4%) and 26.2% (95% CI, 13.9%-42.0%) of participants in the Ad5-nCoV-IH group exhibited a 4-fold or more increase in the median number of spike-specific IgG spots at day 28 and month 6, respectively, which was significantly higher than that of the ZF2001 and CoronaVac groups (**Figure 5B**). Higher concentrations of the RBD-specific IgG in the culture supernatant of R848-activated PBMCs were found in participants boosted with Ad5-nCoV compared to those in ZF2001 or CoronaVac group, and this was consistent with the spike-specific IgG spot response (**Figure 4C**).

### 3.6 Spike-specific IFNγ responses

**Figure 6:**
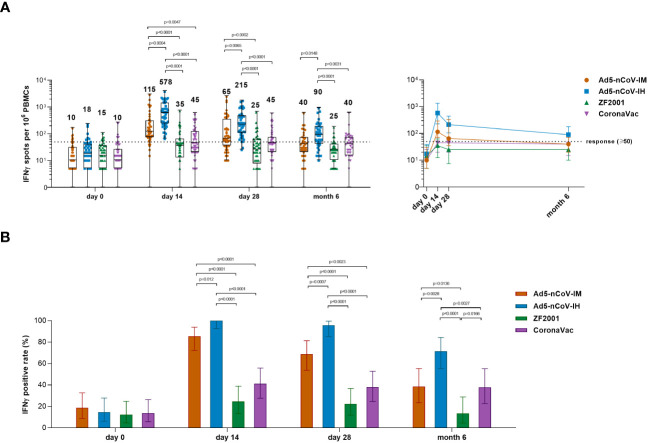
SARS-CoV-2 spike-specific IFNγ ELISpot responses. **(A)** Median numbers of SARS-CoV-2 spike-specific IFNγ spots at days 0 (before booster vaccination), 14, 28 and month 6 after booster vaccination in the four groups. The whiskers indicate the range, the top and bottom of the boxes indicate the interquartile range, and the horizontal line within each box indicates the median. The dashed line indicates the lower limit of the positive response (50 spots per 10^6^ PBMCs). Statistical significance was determined by Kruskal–Wallis test with Dunn’s multiple comparisons tests. **(B)** Percentage of participants with a positive IFNγ response at days 0, 14 and 28 after booster vaccination. Responses were considered positive if there were ≥50 spike-specific spot-forming cells (SFCs) per 10^6^ PBMCs and the ratio of spots in the stimulated wells to spots in background wells was no less than 2.1. Error bars indicate 95% CIs. Statistical significance was determined by two-sided χ^2^ tests or Fisher’s exact test. concentrations for each group, and connecting lines reflect median concentration. Statistical significance was determined by Kruskal–Wallis test with Dunn’s multiple comparisons tests.

Spike-specific IFNγ responses were detected at days 0, 14, 28 and month 6 after booster vaccination to determine the T-cell responses (**Figure 6**). Booster vaccinations induced a rapid spike-specific IFNγ response compared with baseline levels. The participants who received the Ad5-nCoV booster vaccination showed higher T-cell responses than those who received ZF2001 or CoronaVac, and aerosolized Ad5-nCoV booster vaccination induced the greatest IFNγ response (**Figure 6A**). The response was 100% (95% CI, 92.6%-100.0%) and 95.7% (95% CI, 85.2%-99.5%) with the aerosol Ad5-nCoV booster and 85.4% (95% CI, 72.2%-93.9%) and 68.8% (95% CI, 53.7%-81.3%) with the intramuscular Ad5-nCoV booster at days 14 and 28, respectively; both performed better than ZF2001 and CoronaVac (response, <25% for ZF2001 and <42% for CoronaVac) (**Figure 6B**). At month 6, the median number of IFNγ spots per 10^6^ PBMCs was 90 (IQR, 40-179) for Ad5-nCoV-IH, which was significantly stronger than that for Ad5-nCoV-IM (P=0.0148), ZF2001 (P<0.0001) and CoronaVac (P=0.0031). Participants in Ad5-nCoV-IH group also had the highest IFNγ positive response of 71.4%, compared to those in Ad5-nCoV-IM group (38.5%, P=0.0028), ZF2001 group (13.5%, P<0.0001) and CoronaVac group (37.8%, P=0.0027) on 6 month after the booster. All the data suggested that the booster vaccination of aerosolized Ad5-nCoV could induce a strong and durable spike-specific IFNγ response against SARS-CoV-2 in individuals primed with inactivated COVID-19 vaccines.

## 4 Discussion

In China, where more than 95% of individuals vaccinated against COVID-19 received inactivated vaccines, we evaluated the immunogenicity of homologous and heterologous boosters in adults who received prime vaccination with two doses of the inactivated COVID-19 vaccine approximately 6 months prior. Both homologous and heterologous booster vaccination led to an increase in levels of spike RBD-specific binding antibodies, neutralizing antibodies, the B-cell response and T-cell responses from day 14 after booster vaccination, but these increases were highest in participants who received heterologous regimens with an adenovirus-based COVID-19 vaccine.

Booster vaccination with the Ad5-nCoV vaccine induced a superior T-cell response and neutralizing antibody responses compared to those induced by the homologous inactivated vaccine booster or heterologous recombinant protein vaccine booster, regardless of whether intramuscular injection or aerosol inhalation was used (11). At day 7 after booster vaccination, intramuscular Ad5-nCoV induced an obvious IgG antibody response, but no IgG antibody response was found in the aerosolized Ad5-nCoV group, indicating that aerosolized Ad5-nCoV stimulated a slower systemic immune response than that stimulated by intramuscular injection. At day 14 after booster vaccination, the systemic immune advantage of aerosolized Ad5-nCoV was fully demonstrated. Only 1/5 of the dose given by intramuscular injection produced a T-cell immune response higher than that of intramuscular Ad5-nCoV. The binding and neutralizing antibodies against the wild-type strain induced by aerosolized Ad5-nCoV were slightly decreased at day 14 compared to those induced by intramuscular injection, but the level of neutralizing antibodies against the Omicron variant was greater than that induced by intramuscular injection. The neutralizing antibodies from most intramuscular COVID-19 vaccines peak at day 14 after booster vaccination and then decline (24). At day 28 after booster vaccination, the neutralizing antibodies induced by aerosolized Ad5-nCoV still tended to be increased compared to those at day 14, showing different kinetics from other intramuscular booster vaccination regimens examined in this study.

The durability of the humoral response is crucial for COVID-19 vaccines. In our study, participants who received two-dose of CoronaVac 6 months ago showed a low seropositivity rate for neutralizing antibodies against SARS-CoV-2, which is similar to other findings (25, 26). In contrast, the generation of robust and persistent humoral responses in subjects primed with mRNA-based or adenovirus vector-based vaccines has been reported in several trials. A high seropositivity of SARS-CoV-2-specific neutralizing antibody that was elicited by mRNA-1273 or BNT126b2 persisted through 6 months after the second dose (27–29). AZD1222 and Ad26.CoV2.S elicited the neutralizing antibody response that remains higher compared with that in participants who were seronegative at baseline in 3-6 months post-vaccination (30, 31). In another study that focused on the boost strategies for COVID-19 vaccines, heterologous boost regimens induced higher concentrations of neutralizing antibodies compared to the CoronaVac (16). The BNT162b2 elicited the strongest IgG and neutralizing antibody responses, followed by the AZD1222 and Ad26.CoV2.S. Since a substantial difference in humoral response was observed between the aerosolized and intramuscular Ad5-nCoV group, we thought that both the mRNA-base vaccine and the adenovirus vector-based vaccine by mucosal delivery system could be a good choice in eliciting long-lasting antibody responses.

The highly transmissible Omicron variant severely impairs the neutralizing activity of priming two-dose COVID vaccines, with a more than 10-fold reduction compared to that with the wild-type strain (32–34). However, in the present study, the neutralizing activity against Omicron after booster vaccination was ~2~3-fold lower than that against the wild-type strain, regardless of which vaccine was used. In fact, a very small number of participants showed no reduction in neutralizing antibodies against the Omicron variant in this study. A similar pattern of neutralization against the Omicron variant was observed in mRNA vaccine before and after booster vaccination (33, 35, 36). An additional booster vaccine dose generated a more potent, cross-reactive antibody response compared to that induced by the prime vaccination. Repeated antigen exposure improves the affinity maturation of the neutralizing antibodies and increases the potency, breadth and resilience to viral escape mutations of the neutralizing antibodies (37–39).

T-cell immunity is required for viral clearance and supports the generation and maintenance of high-affinity antibodies (40). It has been proved that Adenovirus-vectored COVID-19 vaccines can induce a strong T-cell response (41, 42). In the previous clinical studies of aerosolized Ad5-nCoV, a Th1-biased cellular immune response was observed in vaccinees (11). Here, aerosolized Ad5-nCoV at a lower dose induced a more substantial systemic IFNγ cellular response than intramuscular Ad5-nCoV. We speculate that the resident cellular responses are stronger in the airway and lung than in the blood, since a stronger cellular response in the lungs was observed in mice vaccinated with the intranasal delivery than the intramuscular delivery of adenovirus-vectored COVID-19 vaccine (43). Viral mutations have a less pronounced impact on T-cell immunity than on neutralizing antibody responses, which can limit the impact of individual viral mutations and potentially enhance protection against severe disease from SARS-CoV-2 variants (44–46).

In the present study, intramuscular or aerosolized Ad5-nCoV-induced neutralizing antibodies and T-cell responses after booster vaccination were significantly higher than those from the recombinant RBD dimer vaccine ZF2001. However, ZF2001 booster vaccination induced 2-fold more neutralizing antibodies than the homologous inactivated vaccine booster. Low cellular responses were detected with the aluminum-adjuvanted recombinant protein and inactivated COVID-19 vaccine. In the case of ChAdOx1 nCoV-19 and BNT162b1-primed vaccination, the immune responses upon booster vaccination with the recombinant protein vaccine (NVX-CoV2373) were also inferior to those upon administration of the adenovirus-vectored boosters, including neutralizing antibodies and cellular immune responses (15, 47). Adenovirus-vectored vaccines are a better alternative to booster regimens based on inactivated vaccine-primed vaccination over recombinant protein vaccines in China.

So far, few studies have reported the longevity of antibody response to the Omicron variant boosted by SARS-CoV-2 vaccines. Immune recall by a homologous 3rd dose of mRNA vaccine in COVID-naive vaccinees greatly enhanced great and stable Omicron-specific antibody response (48). Booster vaccination Omicron-specific neutralization antibody response declined 2.8-fold from peak levels between 2 weeks post-3rd dose and 3 months post-3rd dose of mRNA vaccination (49). In our study, the best antibody persistence was observed in the participants boosted with the aerosolized Ad5-nCoV. The fold reduction in median titer of BA.1-specific PNAb at day 28 versus that at month 3 and that at month 6 was 2.0-fold and 6.5-fold, respectively.

There are some limitations to this study. First, this study only evaluated the immune response of different boosters in young male subjects, although similar results have been observed in subjects of different ages and sexes (12, 17). Second, although the saliva IgA response was confirmed in subjects boosted with the aerosolized Ad5-nCoV, whether IgA was exuded from serum or produced by a local mucosal immune response could not be exactly explained. Saliva IgA had also been observed in other parenteral vaccine recipients (50, 51). More assays need to be developed to analyze local mucosal immune responses, including secretory IgA antibodies and local cellular immune responses. Third, there is a lack of phenotypic analysis or functional characterization of antigen-specific B cells and T cells, which will help to better understand the immune response characteristics of different deliveries or different types of COVID-19 vaccines (28, 52, 53). Last, although we assessed the immunogenicity in 6 months after booster vaccination, the efficacy of different booster vaccinations in people primed with 2 doses of inactivated SARS-CoV-2 vaccines remains to be evaluated.

In summary, in the face of waning immunity and the circulation of SARS-CoV-2 variants, the mucosal IgA, neutralizing antibody and T-cell responses were boosted most efficiently with aerosolized Ad5-nCoV in those who received inactivated vaccines as initial doses. Aerosolized Ad5-nCoV booster probably provides a new tool against infection and transmission of the SARS-CoV-2 Omicron variant.

## Data availability statement

The original contributions presented in the study are included in the article/**Supplementary Material**. Further inquiries can be directed to the corresponding authors.

## Ethics statement

The studies involving humans were approved by the Ethics Committee of 305 Hospital of PLA. The studies were conducted in accordance with the local legislation and institutional requirements. The participants provided their written informed consent to participate in this study.

## Author contributions

YS is the principal investigator of this trial. WC, LH, YWL, and KL designed the trials and the study protocol. ZZ and SW drafted the manuscript. WC and LH contributed to critical review and revising of the report. ZZ, SW, YWL, and KL contributed to data interpretation and revising the manuscript. SW led laboratory analyses. SW, ZZ, PF, XS, YW, ZHZ, JX, YL, YHL, MH, GZ, SL, YZ, FS, YY and XFZ were responsible for laboratory analyses. BW was responsible for statistical analysis. TZ and JLZ contributed to study supervision. XWZ, JS, JZ, KF, PWL, PL, and ZQ led and participated in site work, including recruitment, follow-up and data collection. All authors contributed to the article and approved the submitted version.

## Supplementary material

Supplementary Figure 1 RBD-specific IgG reverse cumulative distribution curves. Reverse cumulative distribution curves denote the percentage of participants in each group that reach a different level of antibody concentration at days 0, 7, 14, 28 and months 3 and 6 after the booster.

Supplementary Figure 2 Seroconversion of PNAb response against wild-type SARS-CoV-2 and Omicron variants. **(A, B)** Per-participant factor changes that were calculated by dividing the after-booster response by the before-booster titer for PNAb against wild-type SARS-CoV-2 **(A)** or Omicron BA.1 **(B)** at day 14, 28 and month 3 after the booster vaccination. C Per-participant factor changes that were calculated by dividing the after-booster response by the before-booster titer for PNAb against wild-type SARS-CoV-2, Omicron BA.1 or BA.4/5 at month 6 after the booster vaccination. The whiskers indicate the range, the top and bottom of the boxes indicate the interquartile range, and the horizontal line within each box indicates the median. The dashed line indicates a factor change of 4 (the lower limit of seroconversion), and the number above the dashed line indicates the seroconversion of PNAb responses.

Supplementary Figure 3 Correlation between PNAb and RBD-specific IgG antibodies. **(A)** Spearman’s correlation and linear regression (diagonal lines) analyses were performed with log-transformed data of PNAb titers and RBD-IgG concentrations in all participants. Spearman r and corresponding two-tailed P values are shown in the top left corner. B-C Spearman’s correlation was performed with log-transformed data of wild-type SARS-CoV-2 **(B)** or Omicron BA.1-specific **(C)** PNAb titers and RBD-IgG concentrations in indicated time points and groups. Spearman r values are shown in cells. The stronger the intensity of the cell color is, the stronger the correlation.

Supplementary Figure 4 Comparison of PNAb titers between wild-type SARS-CoV-2 and the Omicron BA.1 and BA.4/5. **(A)** The median titers of PNAb against the wild-type SARS-CoV-2 and the Omicron BA.1 variant in four groups at days 14, 28 and month 3 after the booster vaccination were compared. **(B)** The median titers of PNAb against the wild-type SARS-CoV-2 and the Omicron BA.1 and BA.4/5 variant in four groups at month 6 after the booster vaccination were compared. Black numbers on the top of the bars are median titers for the group. Red numbers on the top are the fold decline in PNAb median concentrations from the wild-type SARS-CoV-2 to the Omicron BA.1 or BA.4/5 variant in each group.

Supplementary Figure 5 Correlates of sVNT NAb titers and PNAb titers. Spearman’s correlation and linear regression (diagonal lines) analyses were performed for each group with log-transformed data of the indicated sVNT NAb titers and PNAb titers at day 28 **(A, B)** and month 6 **(C, D)** after the booster. Spearman r and corresponding two-tailed P values are shown in the top left corner.
