# Supplementary figures and images for: Boosting with an aerosolized Ad5-nCoV elicited robust immune responses in inactivated COVID-19 vaccines recipients
Source: Front Immunol. 2023 Oct 4;14:1239179. doi: 10.3389/fimmu.2023.1239179 (PMC10585368; doi:10.3389/fimmu.2023.1239179)

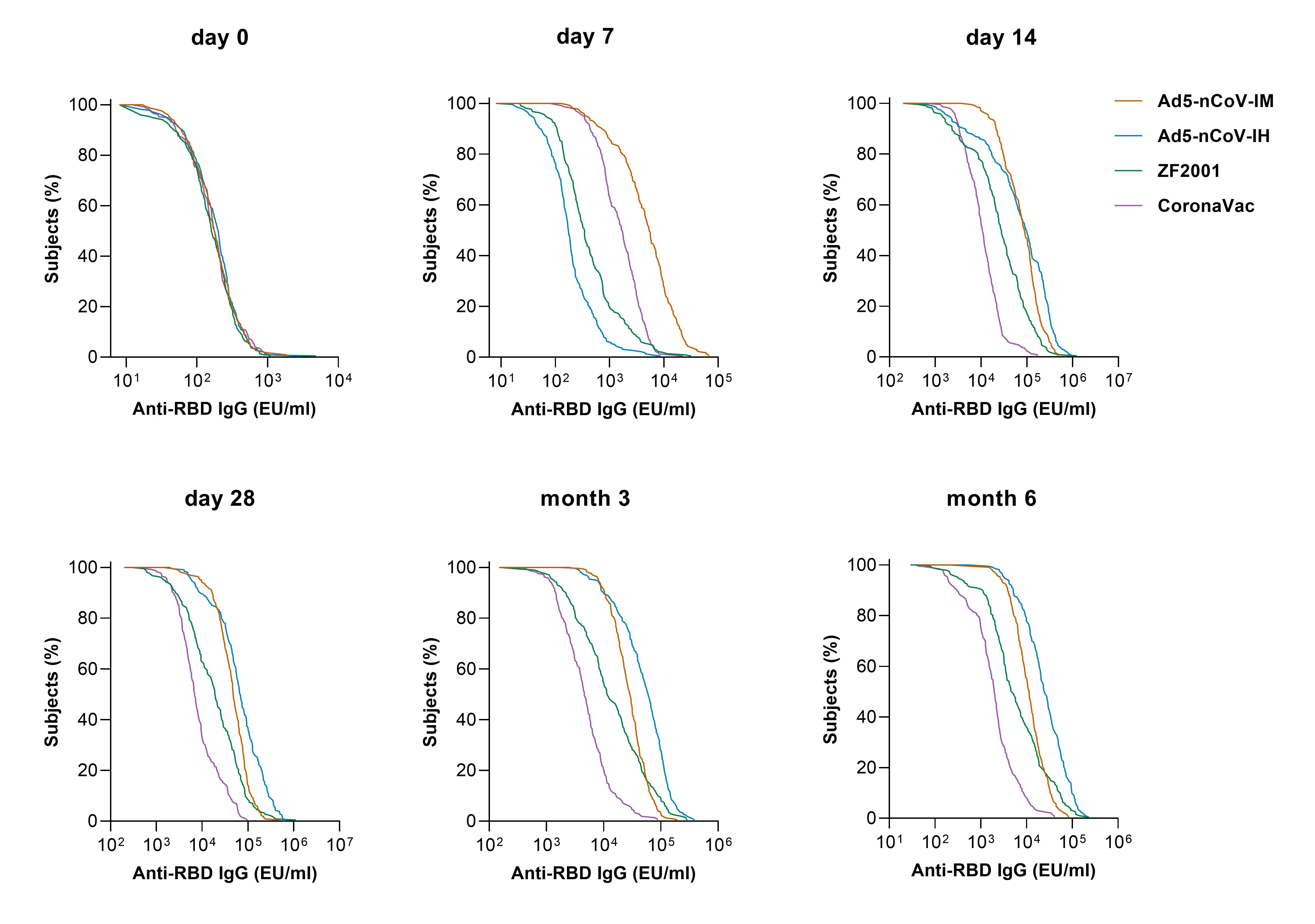

Supplement: Supplementary Figure 1 — RBD-specific IgG reverse cumulative distribution curves. Reverse cumulative distribution curves denote the percentage of participants in each group that reach a different level of antibody concentration at days 0, 7, 14, 28 and months 3 and 6 after the booster. [file Image_1.jpeg]

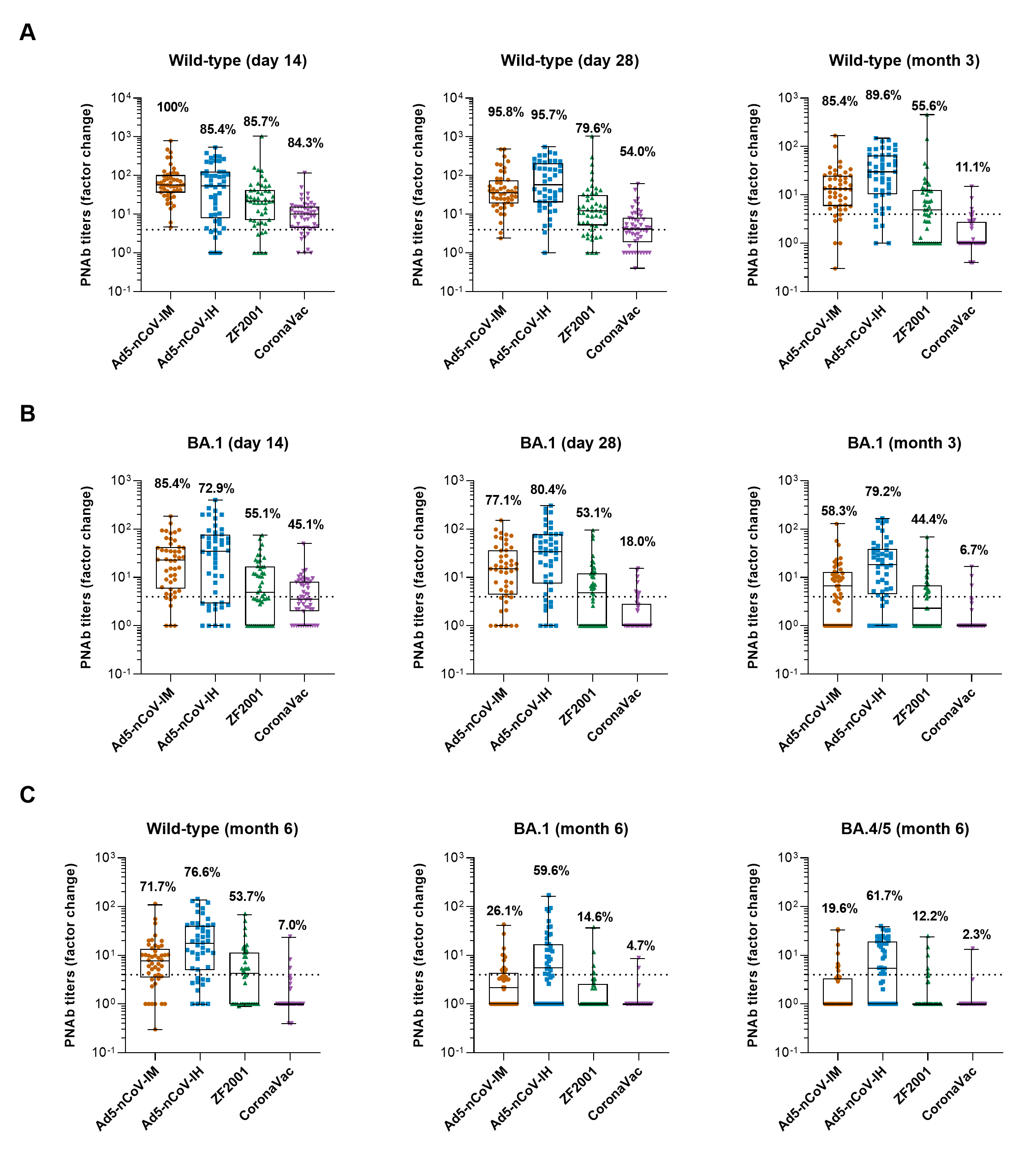

Supplement: Supplementary Figure 2 — Seroconversion of PNAb response against wild-type SARS-CoV-2 and Omicron variants. (A, B) Per-participant factor changes that were calculated by dividing the after-booster response by the before-booster titer for PNAb against wild-type SARS-CoV-2 (A) or Omicron BA.1 (B) at day 14, 28 and month 3 after the booster vaccination. C Per-participant factor changes that were calculated by dividing the after-booster response by the before-booster titer for PNAb against wild-type SARS-CoV-2, Omicron BA.1 or BA.4/5 at month 6 after the booster vaccination. The whiskers indicate the range, the top and bottom of the boxes indicate the interquartile range, and the horizontal line within each box indicates the median. The dashed line indicates a factor change of 4 (the lower limit of seroconversion), and the number above the dashed line indicates the seroconversion of PNAb responses. [file Image_2.tif]

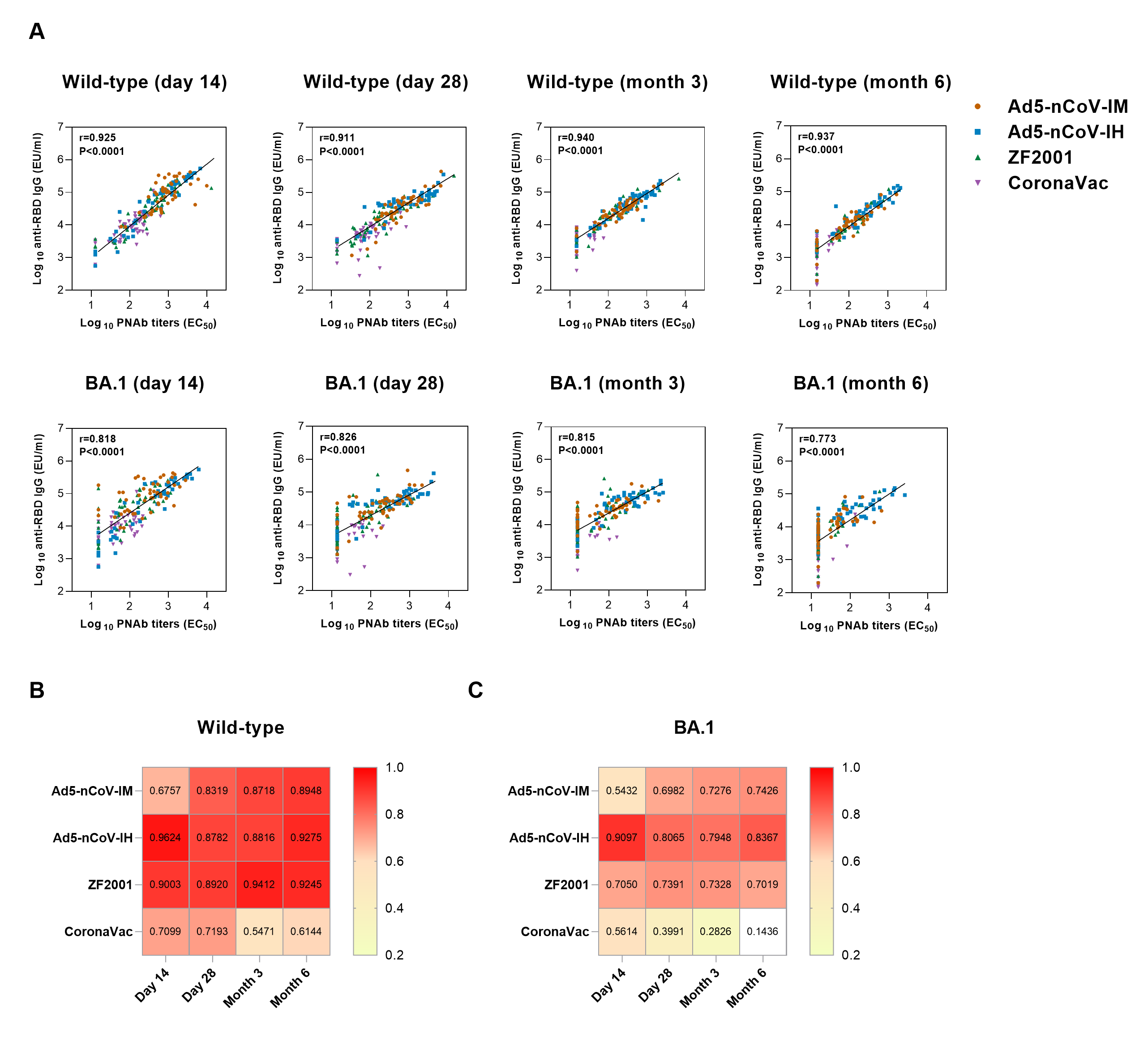

Supplement: Supplementary Figure 3 — Correlation between PNAb and RBD-specific IgG antibodies. (A) Spearman’s correlation and linear regression (diagonal lines) analyses were performed with log-transformed data of PNAb titers and RBD-IgG concentrations in all participants. Spearman r and corresponding two-tailed P values are shown in the top left corner. B-C Spearman’s correlation was performed with log-transformed data of wild-type SARS-CoV-2 (B) or Omicron BA.1-specific (C) PNAb titers and RBD-IgG concentrations in indicated time points and groups. Spearman r values are shown in cells. The stronger the intensity of the cell color is, the stronger the correlation. [file Image_3.tif]

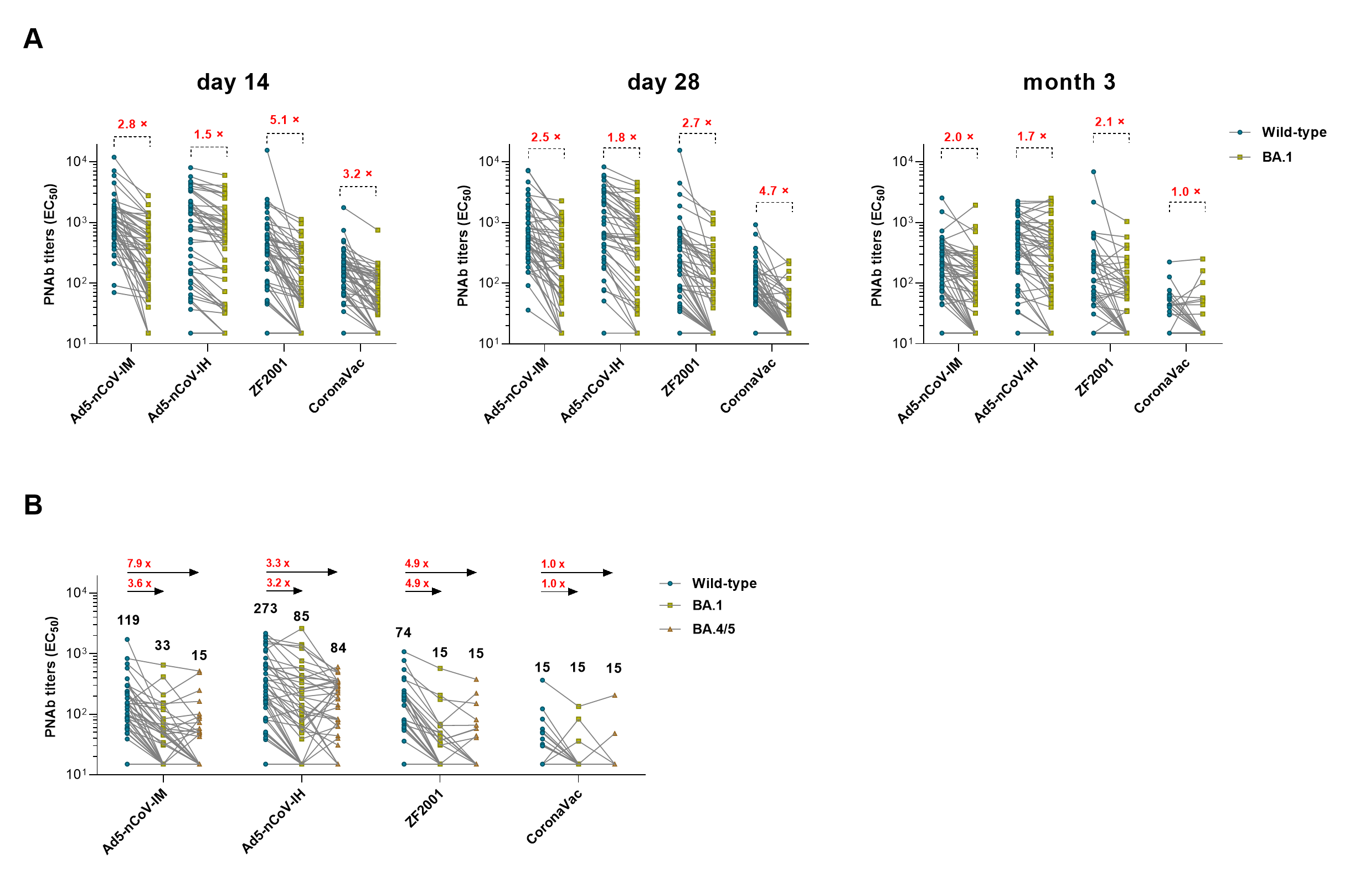

Supplement: Supplementary Figure 4 — Comparison of PNAb titers between wild-type SARS-CoV-2 and the Omicron BA.1 and BA.4/5. (A) The median titers of PNAb against the wild-type SARS-CoV-2 and the Omicron BA.1 variant in four groups at days 14, 28 and month 3 after the booster vaccination were compared. (B) The median titers of PNAb against the wild-type SARS-CoV-2 and the Omicron BA.1 and BA.4/5 variant in four groups at month 6 after the booster vaccination were compared. Black numbers on the top of the bars are median titers for the group. Red numbers on the top are the fold decline in PNAb median concentrations from the wild-type SARS-CoV-2 to the Omicron BA.1 or BA.4/5 variant in each group. [file Image_4.tif]

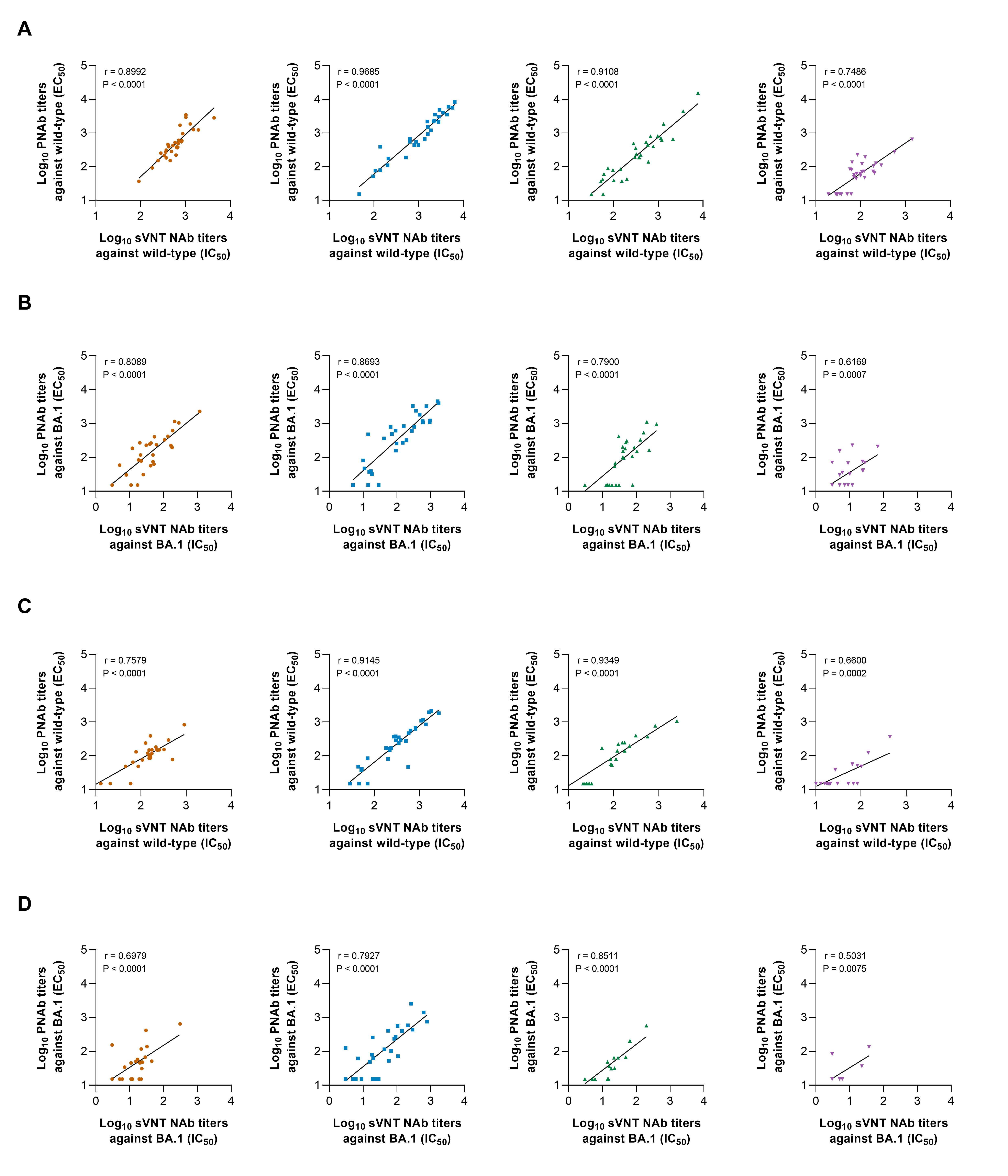

Supplement: Supplementary Figure 5 — Correlates of sVNT NAb titers and PNAb titers. Spearman’s correlation and linear regression (diagonal lines) analyses were performed for each group with log-transformed data of the indicated sVNT NAb titers and PNAb titers at day 28 (A, B) and month 6 (C, D) after the booster. Spearman r and corresponding two-tailed P values are shown in the top left corner. [file Image_5.jpeg]
